# Accuracy of Preoperative Ultrasonographic Airway Assessment in Predicting Difficult Laryngoscopies in Adult Patients

**DOI:** 10.7759/cureus.35652

**Published:** 2023-03-01

**Authors:** Sinchana Bhagavan, Kiran Nelamangala

**Affiliations:** 1 Anaesthesiology, Sri Devaraj Urs Medical College, Kolar, IND

**Keywords:** laryngoscopy, distance from skin to epiglottis midway between the hyoid bone and thyroid cartilage, dsem, minimal distance from the hyoid bone to skin, dshb, preoperative ultrasonography, adult, airway assessment, ultrasonography, difficult laryngoscopy

## Abstract

Background and objectives

Presently, neck ultrasonography is used as a tool to predict a difficult airway. There are no standardized ultrasonographic criteria that help to predict a difficult airway. This study aims to ultrasonographically assess the anterior neck soft tissue thickness preoperatively based on two parameters-the minimal distance from the hyoid bone to skin (DSHB) and the distance from the skin to the epiglottis midway between the hyoid bone and thyroid cartilage (DSEM)-and find out whether these parameters can predict a difficult airway in adults by correlating with the Cormack-Lehane (CL) grading.

Background and objectives

After obtaining ethical committee clearance and patient consent, we conducted this study on 96 patients aged between 18 and 60 years, belonging to American Society of Anesthesiologists (ASA) classes one and two, who were admitted for elective surgery under general anesthesia with endotracheal intubation to RL Jalappa Hospital and Research Centre, Tamaka, Kolar, from January 2020 to May 2021. The exclusion criteria were patients with anticipated difficult airway cases, such as obesity, pregnancy, head and neck anatomical pathologies, maxillofacial anomalies, and edentulous patients. The sonography of the airway was first performed preoperatively by an anesthesiologist along with standard clinical tests such as Mallampati (MP) grading. The sonography included two parameters: DSHB and DSEM. The patients were later classified as having easy or difficult laryngoscopy based on USG criteria from the available literature. A DSHB value of greater than 0.66 cm was predicted to be a difficult airway, and less than 0.66 cm was predicted to be easy. A DSEM value greater than 2.03 cm was predicted to be a difficult airway, and less than 2.03 cm was predicted to be easy.

After induction of anesthesia, another experienced anesthesiologist performed direct laryngoscopy in the sniffing position with an appropriate-sized Macintosh blade and CL grades. CL grades I and II were considered to be easy laryngoscopies.

The quantitative data were presented by mean SD and confidence interval (CI). The qualitative data were presented in percentages, and p-values less than 0.05 were considered statistically significant. To determine the discriminative power of individual tests, the receiver operating characteristic curve and the area under the curve with a 95% confidence interval was noted.

Results

The two USG parameters DSHB and DSEM may be used to predict difficult laryngoscopy in adult patients, as both have very strong statistical significance. Of the two parameters, DSHB seems to have a better diagnostic value for predicting a difficult airway in our study, as supported by the area under the curve (AUC) of 97.4% compared to DSEM with an AUC of 88.8%. DSHB has better sensitivity (100%), and DSEM has better specificity (89.77%).

Conclusion

Our study showed that DSHB and DSEM may aid in predicting difficult laryngoscopies, as a strong statistical significance was present between sonographic measurements and CL grading. DSHB also appeared to have a better diagnostic value for predicting a difficult airway.

## Introduction

Airway management is an important component of clinical anesthesia and involves the maintenance of a patient’s airway to ensure proper gas exchange via mask ventilation or through an airway device [1]. Unpredictable, difficult laryngoscopies can adversely affect ventilation and intubation. Difficult laryngoscopy and intubation are estimated at 1.5%-13% [2]. Among deaths related to anesthesia, 28% occur due to ineffective mask ventilation or an inability to intubate. If a difficult airway is anticipated before induction of anesthesia, proper planning can be made regarding proper equipment and technique, and experienced anesthesiologists can be involved in managing the airway. Various methods can predict a difficult airway, but none of them are completely accurate [3].

The Cormack-Lehane (CL) grading is reliable in predicting a difficult airway, and it is used to grade the difficulty of laryngoscopy. However, this invasive procedure cannot be carried out in conscious patients or for pre-anesthetic airway evaluation in patients with no previous history of endotracheal intubation [1].

With the advancement of technology in hospitals and the availability of portable USG machines, anesthesiologists can use USG as a clinical tool for assessing airways and ruling out difficult airways to prevent a scenario where ventilation and intubation are not possible [3].

Presently, ultrasonography is used as a tool for predicting a difficult airway. However, ultrasonographic criteria for the prediction of a difficult airway are not standardized. Various anatomical parameters can be used to evaluate the ease of endotracheal intubation. However, their measurements are subjective, depending on the observer, and hence have less sensitivity and specificity [3].

Ultrasonography can yield detailed anatomic information and may assist other methods in the prediction of a difficult airway. The anterior soft tissue thickness of the neck can significantly predict difficult intubations and can be an important aid for an anesthesiologist [4].

This study was performed using two USG parameters to predict difficult laryngoscopies in adult patients. These were thicknesses of the anterior neck soft tissue in the transverse view at the level of the hyoid bone, the minimal distance from the hyoid bone to the skin (DSHB), and the thickness of the anterior neck soft tissue in the transverse view at the level of the thyrohyoid membrane, the distance from the skin to the epiglottis midway between the hyoid bone and thyroid cartilage (DSEM).

We applied these two parameters to our population, which is quite different from the Western population with respect to features such as body weight, the thickness of anterior muscle mass, and build. We then documented the reliability of USG in predicting a difficult airway.

The primary objective was to ultrasonographically assess the anterior neck preoperatively based on two parameters. DSHB (thickness of anterior neck soft tissue in transverse view at the level of the hyoid bone (minimal distance from the hyoid bone to the skin)) and DSEM (thickness of anterior neck soft tissue in transverse view at the level of the thyrohyoid membrane (distance from the skin to the epiglottis midway between the hyoid bone and thyroid cartilage)). The secondary objective was to find out if the above two USG parameters can predict difficult airways in adults by correlating with Cormack-Lehane grading.

So far, only a few studies have correlated ultrasonography of the airway with difficult laryngoscopy. Our institution had not carried out any studies on ultrasonography of the airway to predict a difficult airway.

## Materials and methods

We conducted a cross-sectional study on 96 patients admitted for elective surgery under general anesthesia with endotracheal intubation at RL Jalappa Hospital and Research Centre, Tamaka, Kolar, from January 2020 to May 2021 after obtaining ethical approval from the institutional ethics committee (approval no. SDUMC/KLR/IEC/238/2021-22).

Patients aged 18-60 years, having an ASA physical status of one or two, and requiring endotracheal intubation under general anesthesia for elective procedures were included in the study. Patients with a history of difficult intubation, head and neck anatomical pathologies, restricted neck movements, obesity (BMI > 30 kg/m2), pregnancy, maxillofacial anomalies, and edentulousness were excluded.

Sample Size

The sample size was calculated based on the correlation between DSEM and DSHB in a 2014 study, with 90% power, an alpha error of 5%, assuming a population correlation coefficient of 0.5%, and the total sample size calculated as 52 patients [2].

Statistical Analysis

The collected data were coded in an MS Excel spreadsheet, and IBM Statistical Package for Social Sciences (SPSS), version 22, was used to analyze them. The quantitative data were presented as mean +/- standard deviation (SD). Confidence intervals and qualitative data were presented in percentages. P-values less than 0.05 were considered statistically significant. To determine the discriminative power of individual tests, the receiver operating characteristic (ROC) curve and the area under the curve (AUC) with a 95% confidence interval was noted. The ROC curve represents a graphical display of sensitivity and specificity. AUC is a measure to assess the validity of the test. An AUC of one indicates a perfect diagnostic test. Sensitivity, specificity, positive predictive value, and negative predictive value were used to compare categorical data.

Each patient was visited preoperatively, and the procedure was explained to them. Written and informed consent was obtained. All the routine investigations required for a preoperative evaluation were done for the proposed surgery.

An assessment of the airway using USG was conducted preoperatively. The patients were laid down supine with their heads in a neutral position without any support underneath, chin lifted, mouth closed, and tongue touching the floor of the mouth. They were to look straight ahead and make no movements. The linear high-frequency probe was placed in the submandibular area in the midline. The thickness of the anterior soft tissue (transverse view) was obtained at two levels: DSHB and DSEM.

The parameters observed were the thickness of the anterior neck soft tissue in the transverse view at the level of the hyoid bone, DSHB, and the thickness of the anterior neck soft tissue in the transverse view at the level of the thyrohyoid membrane, DSEM.

After clinical and USG airway assessment, the patients were classified as having a difficult or easy laryngoscopy. The criteria for USG parameters were selected based on the available literature. A DSEM value greater than 2.03 cm was predicted to be a difficult airway, and less than 2.03 cm was an easy airway. A DSHB value of greater than 0.66 cm was predicted to be a difficult airway, and less than 0.66 cm was predicted to be an easy airway [5].

On a patient’s arrival in the operating room, an IV line was secured, and the patient was pre-medicated with injections of glycopyrrolate 0.005 mg/kg and fentanyl 2 mcg/kg and preoxygenated for three minutes with 100% oxygen. Induction was done with Inj propofol 2 mg/kg, and neuromuscular blockade was achieved with Inj scoline 2 mg/kg IV. Direct laryngoscopy was performed by an anesthesiologist, using an appropriately sized curved Macintosh blade, and the CL grade was noted. Intubating anesthesiologists with at least three years of experience were not involved in the preoperative sonographic and clinical airway assessments. CL grades I and II were considered easy laryngoscopies. After laryngoscopy, the patient was intubated with an appropriate-sized endotracheal tube and allowed to start with surgery.

## Results

In terms of body mass index distribution, 70 patients (72.9%) were in the 18.5-24.9 kg/m2 group, the next common group being the 25.0-29.9 kg/m2 group, amounting to 22 patients (22.9%) as seen in Table 1.

**Table 1:** Body mass index (kg/m2): frequency distribution of patients studied Kg: kilogram; No: number

Body mass index (Kg/m^2^)	No. of patients	Percentage
< 18.5	4	4.2
18.5–24.9	70	72.9
25.0–29.9	22	22.9
Total	96	100.0

The study showed that the DSHB value was less than 0.66 cm in 79 patients (82.3%) and greater than 0.66 cm in 17 (17.7%) out of the total of 96 patients as cited in Table 2.

**Table 2:** DSHB (cm): frequency distribution of patients studied cm: centimeters; No: number

DHSB (cm)	No. of patients	Percentage
< 0.66	79	82.3
> 0.66	17	17.7
Total	96	100.0

The study showed that the DSEM value was less than 2.03 cm in 90 patients (93.8%) and greater than 2.03 cm in six patients (6.3%), as shown in Table 3.

**Table 3:** DSEM (cm): frequency distribution of patients studied cm: centimeters; No: number

DSEM (cm)	No. of patients	%
< 2.03	90	93.8
> 2.03	6	6.3
Total	96	100.0

Table 4 shows that eight patients were observed, as per CL grading, to have difficult laryngoscopy, of which seven (87.5%) had a DSHB greater than 0.66 cm and four (50%) had a DSEM value of greater than 2.03 cm. The p-values for the association of both DSEM and DSHB with CL grade were smaller than 0.001. This showed a strong statistical significance for a strong association between DSHB, DSEM, and the CL grade.

**Table 4:** Association of DSHB and DSEM in relation to CL grade No: number; CL grade: Cormack and Lehane Grade

Variables	CL grade		Total	p-value
	Easy laryngoscopy	Difficult laryngoscopy		
DSHB (cm)				
< 0.66	78 (88.6%)	1 (12.5%)	79 (82.3%)	< 0.001**
> 0.66	10 (11.4%)	7 (87.5%)	17 (17.7%)	
DSEM (cm)				
< 2.03	86 (97.7%)	4 (50%)	90 (93.8%)	< 0.001**
> 2.03	2 (2.3%)	4 (50%)	6 (6.3%)	
Total	88 (100%)	8 (100%)	96 (100%)	

According to Table 5, DSHB has a better diagnostic value in predicting difficult laryngoscopy (the area under the ROC curve is 97.4%) compared to DSEM, which has an 88.8% area under the ROC curve.

DSHB has better sensitivity (sensitivity of 100%) compared to DSEM (sensitivity of 75%), whereas DSEM has better specificity (specificity of 89.77%) compared to DSHB (specificity of 82.95%).

**Table 5:** ROC curve analysis Diagnostic values based on the area under the curve: 0.9–1.0: excellent test; 0.8–0.9: good test; 0.7–0.8: fair test: 0.6–0.7: poor test; 0.5–0.6: fail AUROC: area under receiver operating characteristic curve; cm: centimeters: ROC: receiver operating characteristic curve

Variables	ROC results in the prediction of difficult laryngoscopy				Cut-off	AUROC	SE	p-value
	Sensitivity	Specificity	LR+	LR-				
DSHB (cm)	100.00	82.95	5.87	0.00	> 0.64	0.974	0.019	< 0.001**
DSEM (cm)	75.00	89.77	7.33	0.28	> 1.98	0.888	0.063	< 0.001**

## Discussion

Securing the airway to establish alveolar ventilation without pulmonary aspiration is an important part of the practice of clinical anesthesia. Even when all measures are taken, adverse respiratory events may occur in the perioperative period, which represents one of the prime causes of clinical malpractice for anesthesia-related incidents. At the time of induction of anesthesia, achieving adequate ventilation or performing endotracheal intubation can be difficult, which may result in a catastrophic situation if intubation or ventilation is not possible. Several risk factors have been recognized to aid in anticipating a difficult airway. They include demographic variables (age, sex, race), body mass index, obstructive sleep apnea, facial abnormalities, Mallampati classes three or four, limited cervical mobility, a small mouth opening, a high-arched palate, and a short thyromental distance and neck circumference. Adhikari S et al. showed that screening tests such as Mallampati (MP) classification, thyromental distance, inter-incisor gap, and neck mobility have poor sensitivity in predicting difficult laryngoscopy [6].

Since the introduction of ultrasound, various medical specialties have incorporated technology into daily practice. Most anesthesia departments now have their own ultrasound machines at their disposal. Initially, USG was used by anesthesiologists to aid in performing regional nerve blocks and securing arterial and central lines. However, newer models are compact and portable and can be incorporated into the operating room. Hence, anesthesiologists have expanded their knowledge of USG techniques into airway evaluation. This may be a useful adjunct to conventional clinical screening tools as it visualizes the real-time anatomy and structures of the airway [7-9].

Many studies have been done after the pilot study by Adhikari S et al [6] on finding the utility of USG to aid airway assessment, comparing and contrasting numerous USG parameters, and using specific USG parameters in head and neck cancers. As airway ultrasound was not routinely performed in our institute for predicting difficult laryngoscopy, we conducted this study to determine whether the two chosen USG parameters (DSHB and DSEM) performed preoperatively can predict difficult laryngoscopy by correlating with CL grading in adult patients.

We first performed a USG of the airway preoperatively and analyzed the standard clinical tests such as Mallampati grading. The USG included two parameters: DSHB (thickness of anterior neck soft tissue in the transverse view at the level of the hyoid bone or the minimal distance from the hyoid bone to the skin) and DSEM (thickness of anterior neck soft tissue in the transverse view at the level of the thyrohyoid membrane or the distance from the skin to the epiglottis midway between the hyoid bone and thyroid cartilage).

We noted that 39.6% of the 92 patients had a Mallampati grade I score, 34.4% had a Mallampati grade II score, and 26% had a Mallampati grade III score. Hence, the anticipated difficult airway, according to the clinical bedside screening test, was found in 26% (25) of the patients. The study showed that DSHB was less than 0.66 cm in 79 patients (82.3%) and greater than 0.66 cm in 17 patients (17.7%), as seen in Table 2. The study showed that DSEM was less than 2.03 cm in 90 patients (93.8%) and greater than 2.03 cm in six patients (6.3%), as shown in Table 3. Forty-three patients (44.8%) had CL grade one, 45 (46.9%) had CL grade two, and eight (8.3%) had CL grade III. Hence, eight patients (8.3%) fell into the category of difficult laryngoscopy.

Our study revealed that out of a total of 25 patients predicted to have difficult intubations as per MP grading, five (20%) had DSHB greater than 0.66 cm and no patients had DSEM greater than 2.03 cm. The p-values for the association of DSHB and DSEM in relation to the MP grade were 0.727 and 0.334, respectively. Hence, there was no statistical significance between the two USG parameters and the MP grade.

Our study revealed that out of eight patients with difficult laryngoscopies as per CL grading, seven (87.5%) had a DSHB value greater than 0.66 cm and four (50%) had a DSEM value greater than 2.03 cm, as shown in Table 4. The p-values for the association of both DSEM and DSHB with CL grade were less than 0.001. Hence, there was a strong statistical significance between the two USG parameters and the CL grade.

A ROC curve analysis was done to compare the two USG parameters, which showed that DSHB has a better diagnostic value in predicting difficult laryngoscopy with an AUC of 97.4% when compared to DSEM with an AUC of 88.8%. DSHB also has better sensitivity (sensitivity of 100%) compared to DSEM (sensitivity of 75%), whereas DSEM has better specificity (specificity of 89.77%) compared to DSHB (specificity of 82.95%), which is shown in Table 5. Hence, the study showed that the USG parameters DSHB and DSEM may aid in predicting a difficult laryngoscopy, supported by the strong statistical significance between the two. DSHB also appeared to have a better diagnostic value in predicting difficult airways.

In their study, Saranya, Raj S, et al. [10] performed a USG airway preoperatively of the anterior neck soft tissue thickness at three levels: the hyoid bone, the thyrohyoid membrane, and the suprasternal notch. They compared this to a CL grade at direct laryngoscopy. The demographic variables were age, sex, and BMI. They found increased thickness at all three levels, which correlated with the increased difficulty of intubation. This was similar to our study, but we had considered only two parameters that showed similar results. On ROC curve analysis, the USG measurement made at the thyrohyoid membrane was found to be very sensitive and specific, with 2.08 cm marking the difference between easy and difficult laryngoscopy. However, in our study, measurement at the hyoid bone had better diagnostic value.

Yadav NK, Rudingwa P, et al. [5] measured the anterior neck soft tissue at the level of the hyoid bone, thyrohyoid membrane, and tongue thickness and compared them to the CL grade at intubation. They performed USG in a neutral and sniffing position, unlike in our study, in which USG was only performed in a neutral position. This produced varied results in USG measurements in both positions, where the cut-off values obtained from the ROC curve for DSHB in neutral and sniffing positions were 0.66 cm and 0.77 cm, respectively, while they were 2.03 cm and 1.9 cm for DSEM in the neutral and sniffing positions, respectively. USG also proved to be better than the clinical parameters (inter-incisor gap, Mallampati grade, neck circumference, and thyromental and sternomental distance). Our study had similar results, but we had considered only Mallampati grade.

Yadav U, Singh R, et al. [11] found the effectiveness of the sonographic parameters ANS-hyoid (anterior neck soft tissue thickness at the level of the hyoid), ANS-VC (anterior neck soft tissue thickness at the level of the vocal cords), and pre-E/E-VC (depth of the pre-epiglottis space to distance from the epiglottis to the midpoint of the distance between the vocal cords), which were different from those chosen by us. Clinical parameters included Mallampati classification, thyromental distance, and hyomental distance, while we considered only Mallampati classification. Their study revealed a significant statistical difference between patients with easy and difficult laryngoscopy, and the highest sensitivity was shown by the ANS-VC and AUC, while the hyomental distance showed the highest specificity.

The results of our study can be supported by the anatomical model described by Greenland et al. [12] and Adnet et al. [13]. Based on this model, the upper airways are shaped by two curves: the oropharyngeal (primary) curve and the pharyngo-glosso-tracheal (secondary) curve. Adequate visualization of the larynx needs both curves to be aligned with the visual axis [12]. A greater skin-to-epiglottis distance can be due to a higher upward concavity of the oropharyngeal or primary curve that lowers the visualization of the glottis [14]. The laryngoscope blade also needs to lift the tissue at the hyoid bone, and hence, a higher CL grade is anticipated with an increase in tissue thickness [5]. Therefore, increasing anterior soft tissue thickness at the level of the hyoid bone and thyrohyoid membrane (DSHB and DSEM) can mean a higher CL grade.

Reddy AV, Aasim SA, et al. [15] stated in their study that ultrasound is a useful tool in airway assessment, considering USG parameters (DSHB, ANS-VC, E-VC, and Pre-E), along with noting the CL grading. They found that the ANS-VC was a probable predictor of difficult intubation compared to other parameters.

## Conclusions

We could conclude that sonographic measurements of DSHB and DSEM can be used to predict difficult laryngoscopies in adult patients as we observed significant statistical significance between the USG measurements and CL grading with a p-value less than 0.001. Of the two parameters, DSHB seems to have a better diagnostic value for predicting a difficult airway in our study, as supported by the AUC of 97.4% compared to DSEM with an AUC of 88.8%. DSHB has better sensitivity (100%), and DSEM has better specificity (89.77%).

Our institute had not conducted any studies on airway ultrasonography to predict a difficult airway. Hence, a combination of sonographic and physical tests may further improve the diagnostic value of identifying cases of difficult intubation.
